# Land use change affects water erosion in the Nepal Himalayas

**DOI:** 10.1371/journal.pone.0231692

**Published:** 2020-04-15

**Authors:** Devraj Chalise, Lalit Kumar

**Affiliations:** 1 School of Environmental and Rural Science, University of New England, Armidale, NSW, Australia; 2 Nepal Agricultural Research Council, Chitwan, P.O.B., Nepal; Imperial College London, UNITED KINGDOM

## Abstract

Soil erosion is a global environmental threat, and Land Use Land Cover Changes (LUCC) have significant impacts on it. Nepal, being a mountainous country, has significant soil erosion issues. To examine the effects of LUCC on water erosion, we studied the LUCC in Sarada, Rapti and Thuli Bheri river basins of Nepal during the 1995–2015 period using the Remote Sensing. We calculated the average annual soil loss using the Revised Universal Soil Loss Equation and Geographical Information System. Our results suggest that an increase in the agricultural lands at the expense of bare lands and forests escalated the soil erosion through the years; rates being 5.35, 5.47 and 6.03 t/ha/year in 1995, 2007 and 2015, respectively. Of the different land uses, agricultural land experienced the most erosion, whereas the forests experienced the least erosion. Agricultural lands, particularly those on the steeper slopes, were severely degraded and needed urgent soil and water conservation measures. Our study confirms that the long term LUCC has considerable impacts on soil loss, and these results can be implemented in similar river basins in other parts of the country.

## Introduction

Soil erosion is a severe ecological issue that humanity is facing [1] as it washes away the fertile topsoil, deteriorates soil quality and increases the soil sediments in stream channels [2]. Extensive use of available land for agriculture increases the soil loss at a global scale [3]. It is one of the major environmental issues of hill and mountain ecosystems [4]. Soil erosion is a significant feature in Nepalese terrain, given the hilly topography and rugged mountains, much-concentrated rainfall events in the monsoon (June-September) and increased human influence in the removal of natural vegetation [5, 6]. Numerous studies have been performed to assess the soil erosion in Nepal, mostly in the Middle Mountain region [7] but only a few have addressed the erosion in the High Himalayas [4, 8, 9]. Variation in topography, slope, land use patterns and population pressure across different physiographic regions produces different rates of soil erosion in Nepal, ranging from zero in the lowland areas to 420 t/ha/year in the shrublands [10]. Soil erosion rates of 11.17 and 10.74 t/ha/year have been reported in the Aringale Khola Watershed and Sarada river basin, respectively in the Siwalik Hills [11, 12]. Erosion due to natural causes is the highest in Nepal, but there have been significant impacts of human influence as well on soil erosion [13]. Heavy rainfall events with high-speed winds and hailstorms during the pre-monsoon make soil erosion more problematic in rainfed agriculture in Nepal [14].

Land use is a socioeconomic response that humans exploit to meet their needs [15]. Land Use Land Cover Change (LUCC) was reported to be one of the dominant factors affecting soil erosion in a landscape [16]. LUCC, combined with several atmospheric and topographical conditions, has an accelerating impact on soil and land degradation [17], including acidification, alkalization, soil erosion and nutrient leaching. In recent times, impacts of LUCC and soil erosion have been established to be a critical environmental concern, and significant impacts of the long term LUCC on nutrient losses and sedimentation have been reported at worldwide scale [15, 18–20]. Land use activities such as agricultural crop production accelerate the soil erosion process, ultimately degrading the water quality of streams owing to the accumulation of soil sediments in the water bodies [21]. One of the leading causes of land use change in developing countries is the high rate of physical growth of city areas and extensive agricultural practices in the available land resources [19]. Since the long term changes in land use land cover (LULC) and the climate are also inevitable in the future, it is necessary to investigate the expected impacts of these changes on soil erosion at the catchment scale [17]. LUCC can be due to natural events as well as a result of human activities, and the information derived from the LULC can be used to analyse the effects of LUCC on soil erosion, particularly those that have occurred due to human interventions [22].

Several studies have been undertaken to assess the effects of the LUCC on soil erosion [23, 24]. A 46% increase in soil erodibility was reported in a land use change from grasslands to agriculture in Cankiri-Indagi Mountain Pass, Turkey [25]. Converting grasslands and forests to agricultural lands was found to decrease soil organic carbon, thereby increasing soil erodibility in an experiment conducted in the Southeast of Spain [26]. LUCC may alter the river courses and change the fluvial regime, ultimately modifying the detachment, passage and depositional processes [27] and in the long term, it may change the distribution of soil particles and textural organization [25]. One of the best ways to measure the impacts of LUCC in soil erosion is to use time-series satellite images to analyze the long term LUCC in relation to erosion potential of catchments [2].

This paper aims to assess the soil erosion dynamics induced by the long term LUCC in three central river basins of western Nepal, namely Sarada, Rapti and Thuli Bheri. This research covers all the physiographic regions of Nepal and encompasses dense and open forests, snowy and rugged mountains, agricultural lands, built-up areas, bare lands, water bodies, and public and private road networks. The contributions of these river basins in terms of various ecological and environmental functions are manifold. Poor land use management practices coupled with undulant topography and much erratic rainfall events are the primary drivers of soil erosion in the study area [12, 28]. Farmers in the region are experiencing reduced agricultural productivity owing to the loss of fertile topsoil [12]. Therefore, we can confidently assume that it is going to get worse. Thus, it is essential to examine how soil erosion has changed through the years so that proper soil and water conservation measures can be adopted concentrating more on the affected areas. Land use planners and policymakers may benefit from the results produced to develop proper plans to reduce soil erosion and safeguard natural resources.

## Materials and methods

### Study area and data

**Fig 1:**
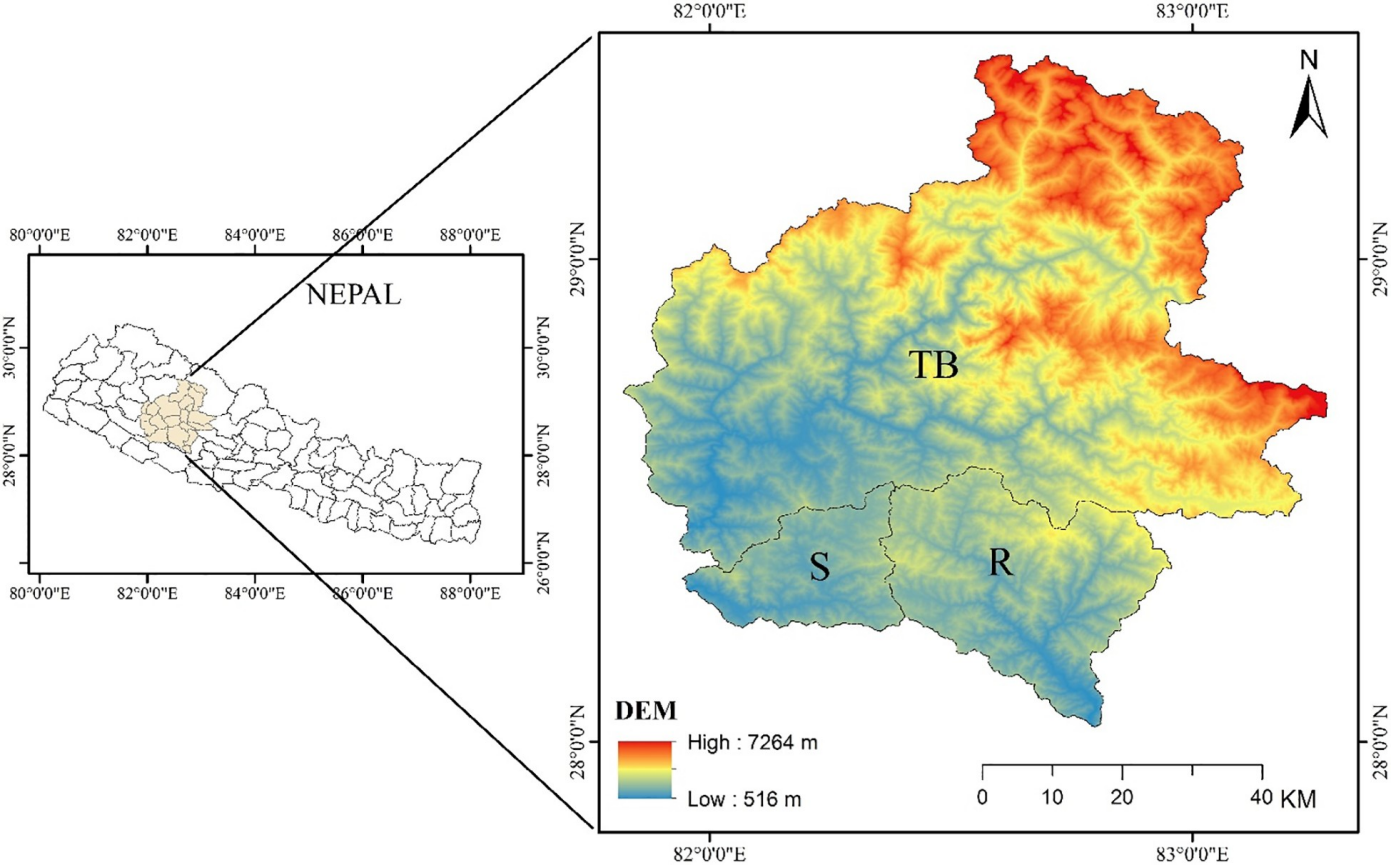
Study area covering the three river basins with the DEM (TB = Thuli Bheri, S = Sarada, R = Rapti).

This study covers an area comprising of three major river basins, namely Thuli Bheri, Sarada and Rapti river basins (Fig 1). The study area lies between 28°1'51'' and 29°25'20'' N latitudes and 81°49'17'' and 83°16'49'' E longitudes and has an area of 12,182 km^2^ (Table 1). It encompasses five distinct physiographic regions of Nepal: Terai Plains (516–700 m), Siwalik Hills (700–1,500 m), Middle Mountains (1,500–2,700 m), High Mountains (2,700–4,000 m) and High Himalayas (4,000–7,264 m) and covers the area of Jajarkot, Rukum and Rolpa districts and some parts of Surkhet, Dang, Pyuthan, Salyan, Baglung, Myagdi, Dolpa, Dailekh, Kalikot, Jumla and Mugu districts. Of the three river basins, Thuli Bheri is the most diverse as it encompasses all the physiographic regions available, with elevation ranging from 523 m in the South to 7,264 m in the North. It is also the largest river basin amongst the three, covering an area of 9,338 km^2^ followed by Rapti and Sarada river basins with 1,972 and 872 km^2^, respectively.

**Table 1:** Location and coverage of the study area.

River basin	Elevation (m)	Location (^0^)	Slope range (^0^)	Physiographic region	Climate	Area (km^2^)
Sarada	521–2,776	81°56'33''– 82°24'13'' E 28°13'45''– 28°32'21'' N	0–65.06	Terai Plains, Siwalik Hills, Middle Mountains and High Mountains	Humid tropical, moist subtropical, temperate and cool to sub-alpine	872
Rapti	516–3,597	82°21'4''– 82°57'22'' E 28°1'51''– 28°34'18'' N	0–71.86	Terai Plains, Siwalik Hills, Middle Mountains and High Mountains	Humid tropical, moist subtropical, temperate and cool to sub-alpine	1,972
Thuli Bheri	523–7,264	81°49'17'' –83°16'49'' E 28°21'5'' - 29°25'20'' N	0–77.43	Terai Plains, Siwalik Hills, Middle Mountains, High Mountains and High Himalayas	Humid tropical, moist subtropical, temperate, cool to sub-alpine and alpine to arctic	9,338

The data used in the analysis are the rainfall measurements (1980–2016) from 53 rainfall stations (Please see S1 File) obtained from the Department of Hydrology and Meteorology, Nepal, and the satellite images from the United States Geological Survey (USGS) Earth Explorer data portal (https://earthexplorer.usgs.gov/). A Digital Elevation Model (DEM) of 20 m resolution was obtained from Nepal Agricultural Research Council, and the soil data were acquired from National Land Use Project of Ministry of Land Reform and Management, Kathmandu, Nepal.

### LUCC analysis

Three satellite imageries covering path 143 and row 40 were used in this study to examine the LUCC dynamics: the Landsat Thematic Mapper (TM) of 30 m spatial resolution acquired on 20 March 1995 and 05 March 2007, and the Landsat Operational Land Imager (OLI) of 30 m spatial resolution acquired on 11 March 2015. The Landsat TM has seven bands, and OLI has 11 spectral bands. LULC were categorized into forests, snow, agriculture, water bodies, built-up area and bare lands (rocky outcrops with no vegetation and riverbanks with sand). The LUCC analysis was done using ENVI version 5.4.

For all the images (TM and OLI), radiometric calibration and Fast Line-of-sight Atmospheric Analysis of Hypercubes atmospheric correction were performed to remove the atmospheric influence, and dark object subtraction method was used to remove the effects of smoke, dust and haze [29]. The LULC maps were developed using the Maximum Likelihood Classification (MLC) because of its strong theoretical base and simplicity [29]. MLC is one of the most preferred parametric techniques as it calculates the likelihood of an unknown vector based on the highest probability of fit based on Bayesian equation [29, 30]. Accuracy assessment of all the classified images was carried out using the points collected from the satellite images. High-resolution satellite images were available for 2007 and 2015 (QuickBird and WorldView) dataset; however, the 1995 dataset did not have high-resolution images. Therefore, the Google Earth image information was supplemented through the author's knowledge of the area as well as expert input. The overall producer and user accuracies were obtained for each classified image, and the Kappa coefficient [31] was calculated to check the accuracy of classified images.

### Soil erosion estimation

We used the RUSLE model [32] to calculate the average soil loss in the study area using the following equation:

E=R×K×LS×C×P

Where *E* is the estimated average soil erosion (t/ha/year), *R* is the rainfall factor (MJ mm/ha/h/year), *K* is the soil erodibility factor (t ha/MJ/mm), *LS* is the combined slope length and slope steepness factor (dimensionless), *C* is the cover management factor (dimensionless), and *P* is the support practice factor (dimensionless).

The *R* factor represents the potential erosivity of soil [33] and relies on the intensity and volume of rainfall occurring in a place over a specified period [34]. The *R* factor was estimated using the following equation [35]:

R=38.5+0.35r

Where r is annual rainfall in mm

The *K* factor represents the soil susceptibility to detachment caused by the beating action of rainfall and runoff water [32, 36]. We acquired soil textural maps of the study area from the National Land Use Project, Nepal, and prepared the *K* factor map assigning values to soil texture as proposed by [37] (Table 2).

**Table 2:** K values for different soil textural classes in the study area.

Soil texture	*K* factor
Clay, clay loam, loam, sandy clay loam, silty clay	0.035
Loamy sand, sand	0.007
Sandy loam	0.018
Silty clay loam, silty loam	0.045

The slope length (*L*) and slope steepness (*S*) are the topographic factors, and they account for the effects of slope length and steepness on soil loss [36]. We calculated the *LS* factor using the following equation given by Wischmeier and Smith [38] and Šurda, Šimonides [39].

$$LS={\left(\frac{Cell\;size}{22.13}\right)}^{m}\times (0.0138+0.0097\;s+0.00138\;s^{2})$$

Where Cell size = Grid cell size (20 m for this study), m = 0.2 to 0.5 (0.2 for slopes less than 1%, 0.3 for 1–3%, 0.4 for 3–4.5% and 0.5 for slopes exceeding 4.5%) [40] and ‘*s*’ is the slope in percentage.

The *C* factor represents the relationship between erosion on bare land and erosion on cultivated land [41] whereas the *P* factor represents the consequences of soil protection measures such as contouring and terracing [12]. The *C* and *P* values were assigned as per the land uses in the vector format [42–45] (Table 3) and converted to raster format to be used with other factor layers. The *K* and *LS* factors being the same for the study years, R factor maps for 1995, 2007 and 2015 were prepared separately using average rainfall data of 1990–2000, 2004–2010 and 2014–2016, respectively. Similarly, individual *C* and *P* factors were developed for 1995, 2007 and 2015 as per the land use map of the study area.

**Table 3:** *C* and *P* values for different LULCs.

LULC	*C* value	*P* value
Agriculture	0.63	0.5
Bare land	0.09	0.7
Built-up area	0.09	1
Forest	0.003	0.8
Snow	0	0
Water bodies	0	0

**Fig 2:**
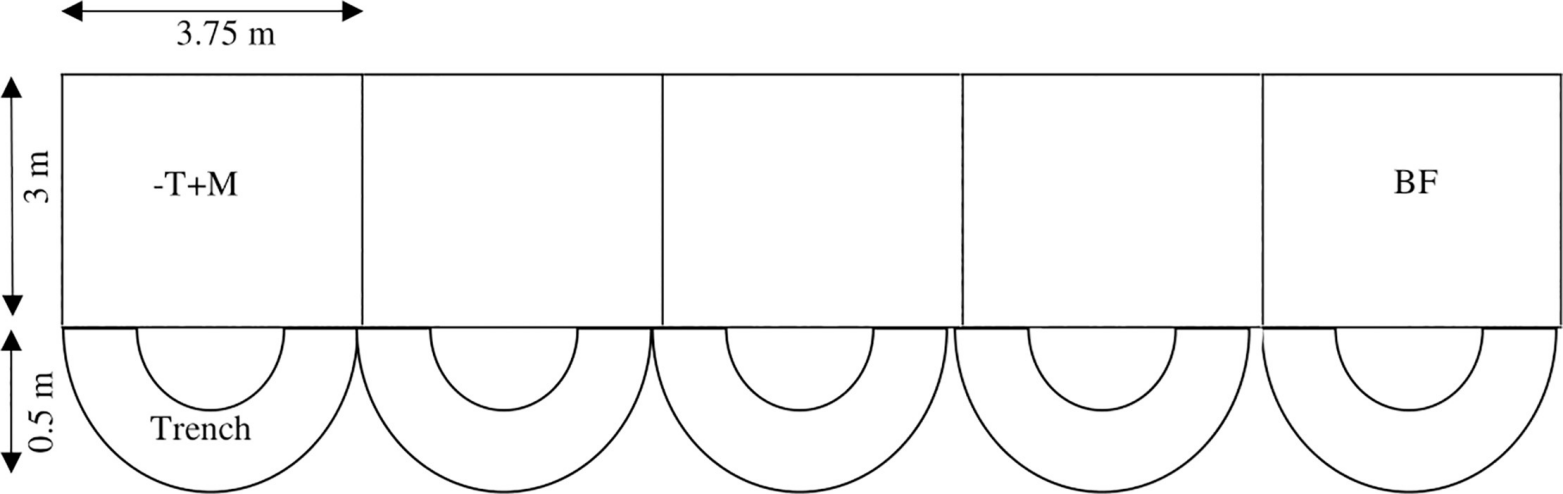
Soil erosion experimental plots (T = Tillage, M = Mulch and BF = Bare Fallow).

We have also established corn and bare fields as soil erosion experimental plots in the study area to facilitate the comparison of the soil erosion rates computed from the RUSLE modelling. Replicated four times each, five treatments: no-tillage + mulch, no-tillage + no mulch, tillage + mulch, tillage + no mulch and bare fallow each with 3 × 3.75 m plot size, were established in an unbalanced complete randomized block design (Fig 2). Manakamana-3, a popular corn variety in the Nepalese hills, was sown with a spacing of 75 cm × 25 cm, and trench of 50 cm was dug at the lower end of experimental plots to collect the soil runoff. Corn was grown in all the treatments except in the bare fallow for two years. The primary objective of this experiment was to assess the impacts of tillage and mulch on soil erosion and corn yield.

### Spatial interpolation

Kriging, a linear geostatistical interpolation technique, was used to prepare the digital map layers for *R* and *K* factors of the RUSLE. The acquired data were fitted to spherical semivariogram and then interpolated with the ordinary kriging technique in ArcMap. Kriging is a vital tool to estimate values at non-sampled places based on the sampled data [46, 47]. It is an interpolation method which approximates the value of a function at a given point as a weighted amount of the function values at the adjacent points [48].

## Results

### LUCC dynamics

**Fig 3:**
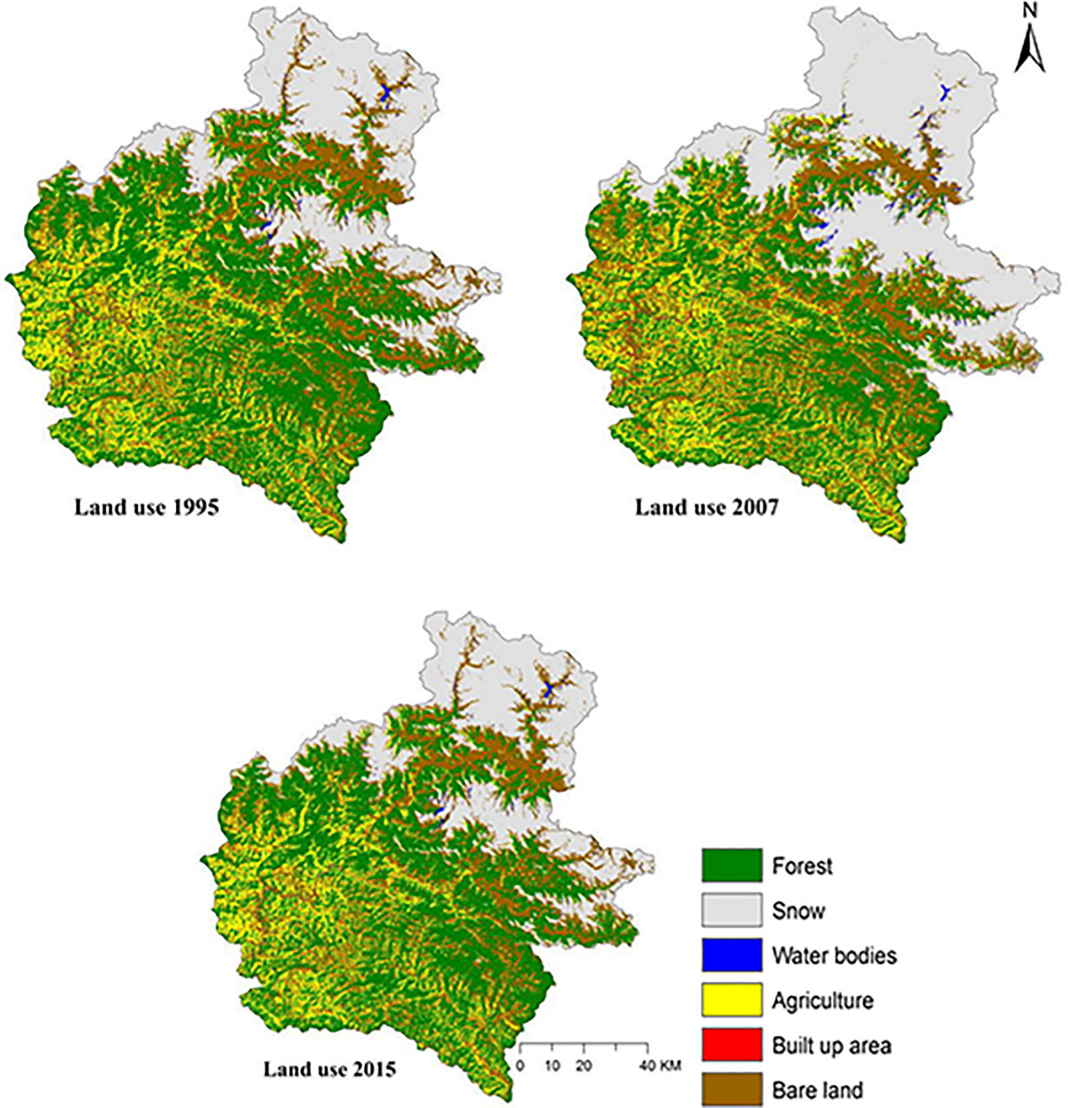
Land use maps for the years 1995, 2007 and 2015.

**Fig 4:**
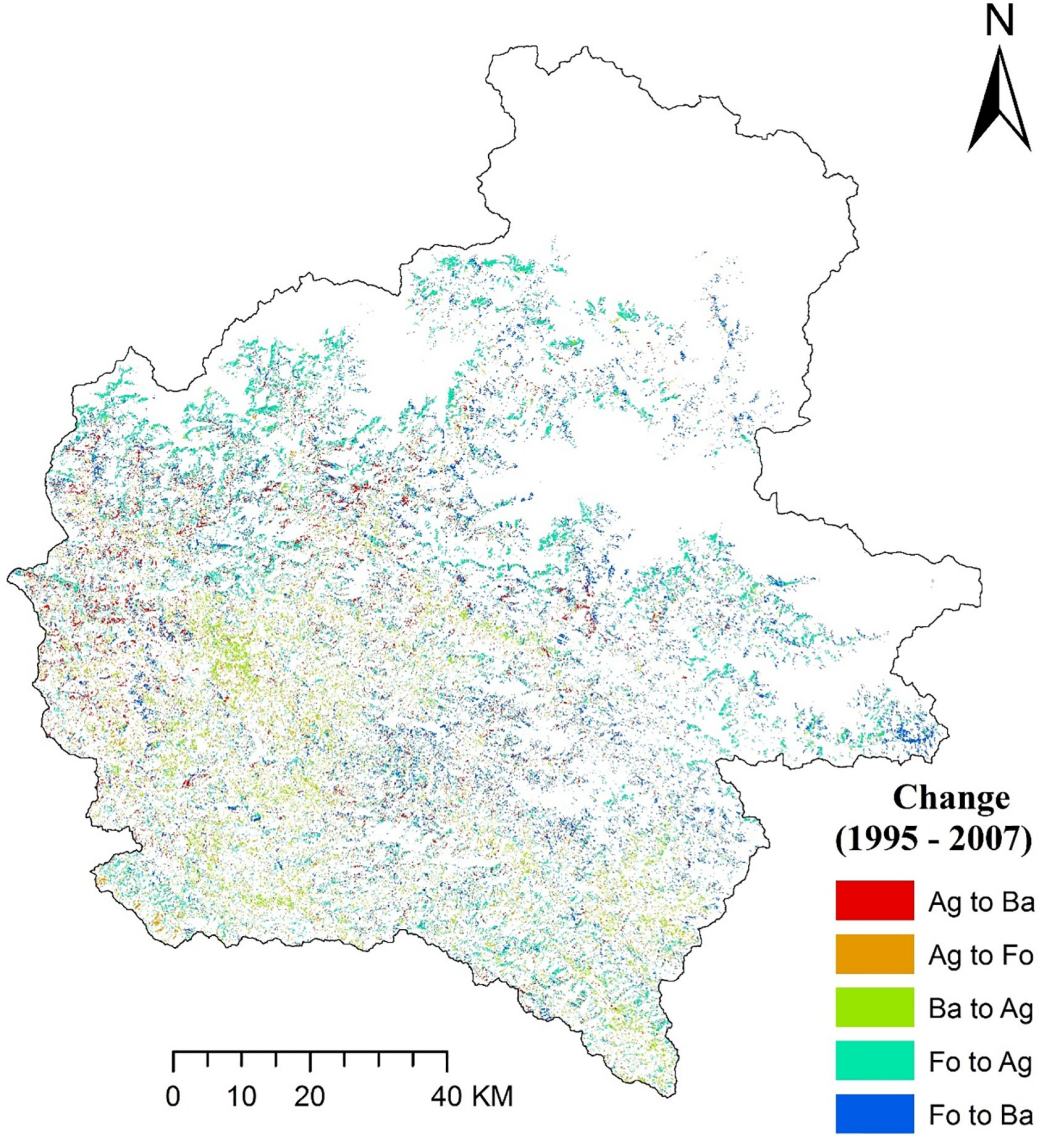
Change analysis thematic map showing land use conversion from 1995 to 2007 (Ag = Agriculture, Ba = Barren land, Fo = Forest).

**Fig 5:**
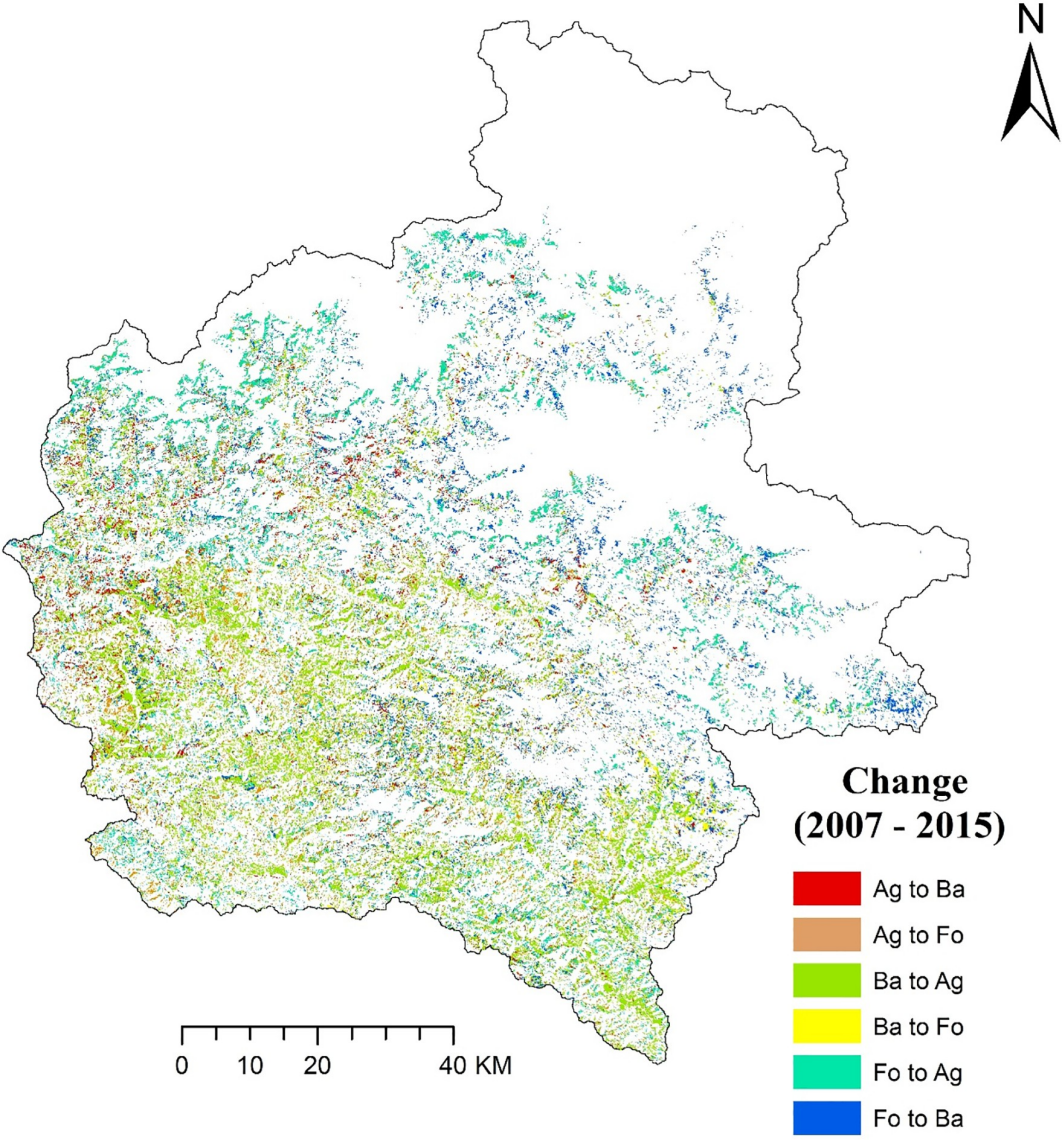
Change analysis thematic map showing land use conversion from 2007 to 2015 (Ag = Agriculture, Ba = Barren land, Fo = Forest).

Our classification accuracies were found to be 85% for 1995, 86% for 2007 and 84% for 2015 with Kappa Coefficient of 0.81, 0.82 and 0.79 for the year 1995, 2007 and 2015, respectively. The relative proportion of different LULC classes during the study periods and percentage change in LULC through the years are shown in Table 4 and Fig 3. The land use change matrix of the study area is presented in Table 5, and change analysis thematic maps showing land use conversion from 1995 to 2007 and 2007 to 2015 are presented in Figs 4 and 5, respectively. A detailed analysis of satellite imageries revealed varying degrees of changes in the composition of LULC categories over 21 years. Forests, agriculture, bare lands and snow were the most prominent LULC, and they covered nearly 95% of the study area. Water bodies and bare lands had experienced continuous decline through the years whereas the agricultural and built-up area had increased. Our study shows that forest area reduced during the period 1995–2007; however, between 2007 and 2015, the forest area increased. Areal coverage of forest was the maximum in the study area; for all the study periods with areas of 4,695, 3,518 and 4,171 km^2^ in the years 1995, 2007 and 2015, respectively. The built-up area covering only 0.2 km^2^ of the study area in 1995 has increased exponentially to 7.1 and 16.1 km^2^ in 2007 and 2015, respectively. Water bodies had a significant decline of 60% during the period of 1995–2015. With the increasing population, human settlements have moved up to steeper slopes and higher elevations; thus, aggravating the risk of soil erosion through the years.

**Table 4:** Distribution and percent change in LULC through the years 1995, 2007 and 2015.

LULC	Area						Percent change in area		
	1995		2007		2015		1995–2007	2007–2015	1995–2015
	Area (km^2^)	(%)	Area (km^2^)	(%)	Area (km^2^)	(%)			
Agriculture	1780.78	14.61	2178.56	17.89	2722.3	22.35	22.33	24.95	52.87
Bare land	2994.56	24.59	2504.96	20.56	2311.5	18.97	-16.34	-7.72	-22.81
Built-up area	0.2	0.001	7.1	0.05	16.1	0.13	3450	126.76	7950
Forest	4695.26	38.54	3518.04	28.88	4170.76	34.24	-25.07	18.55	-11.17
Snow	2673.3	21.95	3942.44	32.37	2946.44	24.19	47.47	-25.26	10.21
Water bodies	38.2	0.31	31.2	0.25	15.2	0.12	-18.32	-51.28	-60.21

**Table 5:** Land use change matrix of the study area (1995–2007 and 2007–2015) (Area in km^2^).

**2007**	**1995**						
	**Agriculture**	**Bare land**	**Built-up area**	**Forest**	**Snow**	**Water bodies**	**Total**
**Agriculture**	1312.62	326.42	0	538.82	0.6	0.1	2178.56
**Bare land**	236.12	1819.62	0	443.62	1.1	4.5	2504.96
**Built-up area**	1.4	4.7	0.2	0.8	0	0	7.1
**Forest**	116.32	59.2	0	3340.82	0.9	0.8	3518.04
**Snow**	114.22	780.12	0	354.5	2670.4	23.2	3942.44
**Water bodies**	0.1	4.5	0	16.7	0.3	9.6	31.2
**Total**	1780.78	2994.56	0.2	4695.26	2673.3	38.2	12182.3
**2015**	**2007**						
	**Agriculture**	**Bare land**	**Built-up area**	**Forest**	**Snow**	**Water bodies**	**Total**
**Agriculture**	1633.06	829.2	5	179.14	75.8	0.1	2722.3
**Bare land**	71.9	1333.7	1.4	64.1	831.9	8.5	2311.5
**Built-up area**	2.1	3	0.1	0.1	10.8	0	16.1
**Forest**	471.1	334.56	0.6	3268.8	88.4	7.3	4170.76
**Snow**	0.2	2.2	0	3.9	2934.14	6	2946.44
**Water bodies**	0.2	2.3	0	2	1.4	9.3	15.2
**Total**	2178.56	2504.96	7.1	3518.04	3942.44	31.2	12182.3

### RUSLE factors and soil erosion maps

**Fig 6:**
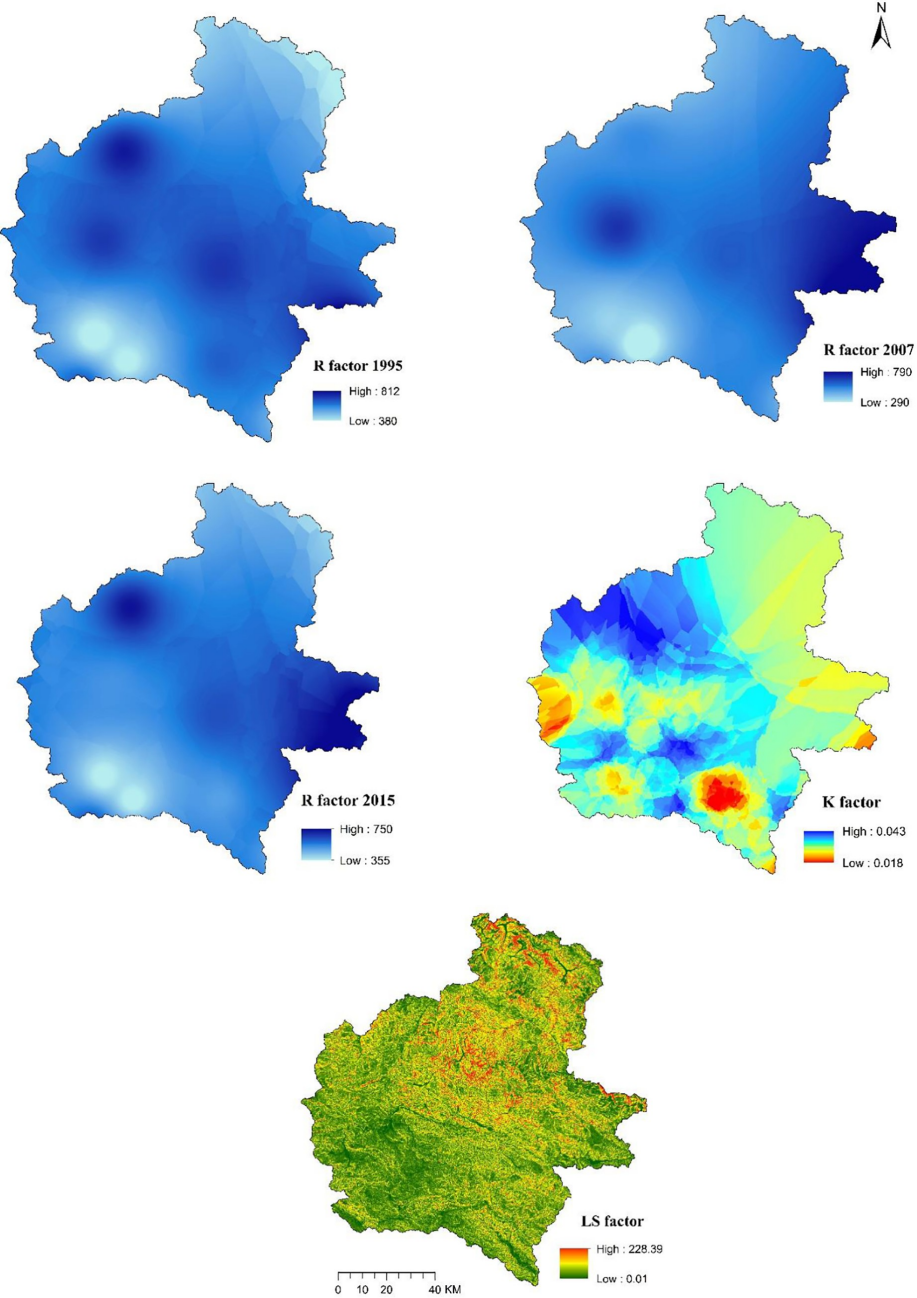
Spatial distribution of R, K and LS factor.

**Fig 7:**
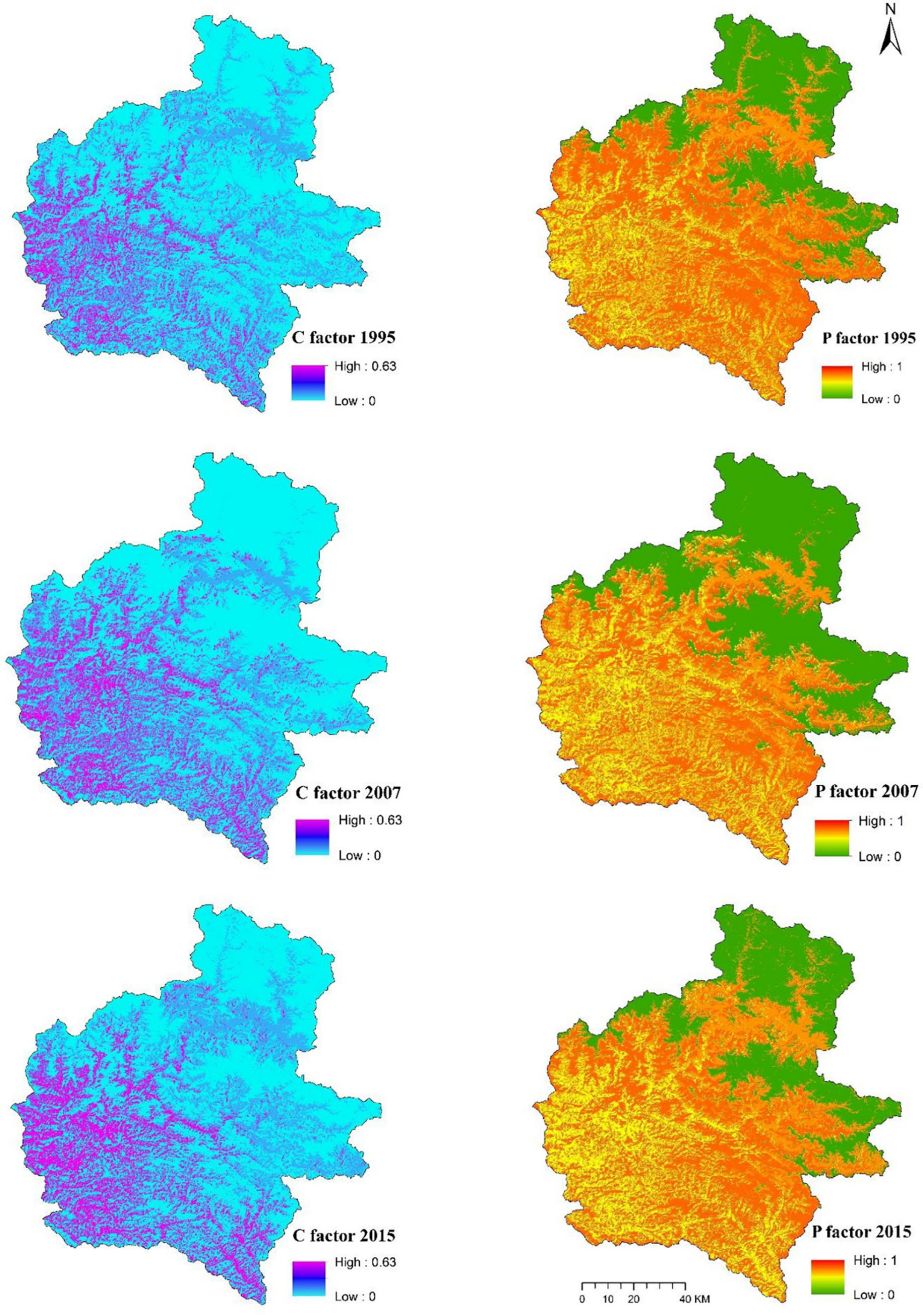
Spatial distribution of C and P factors for 1995, 2007 and 2015.

**Fig 8:**
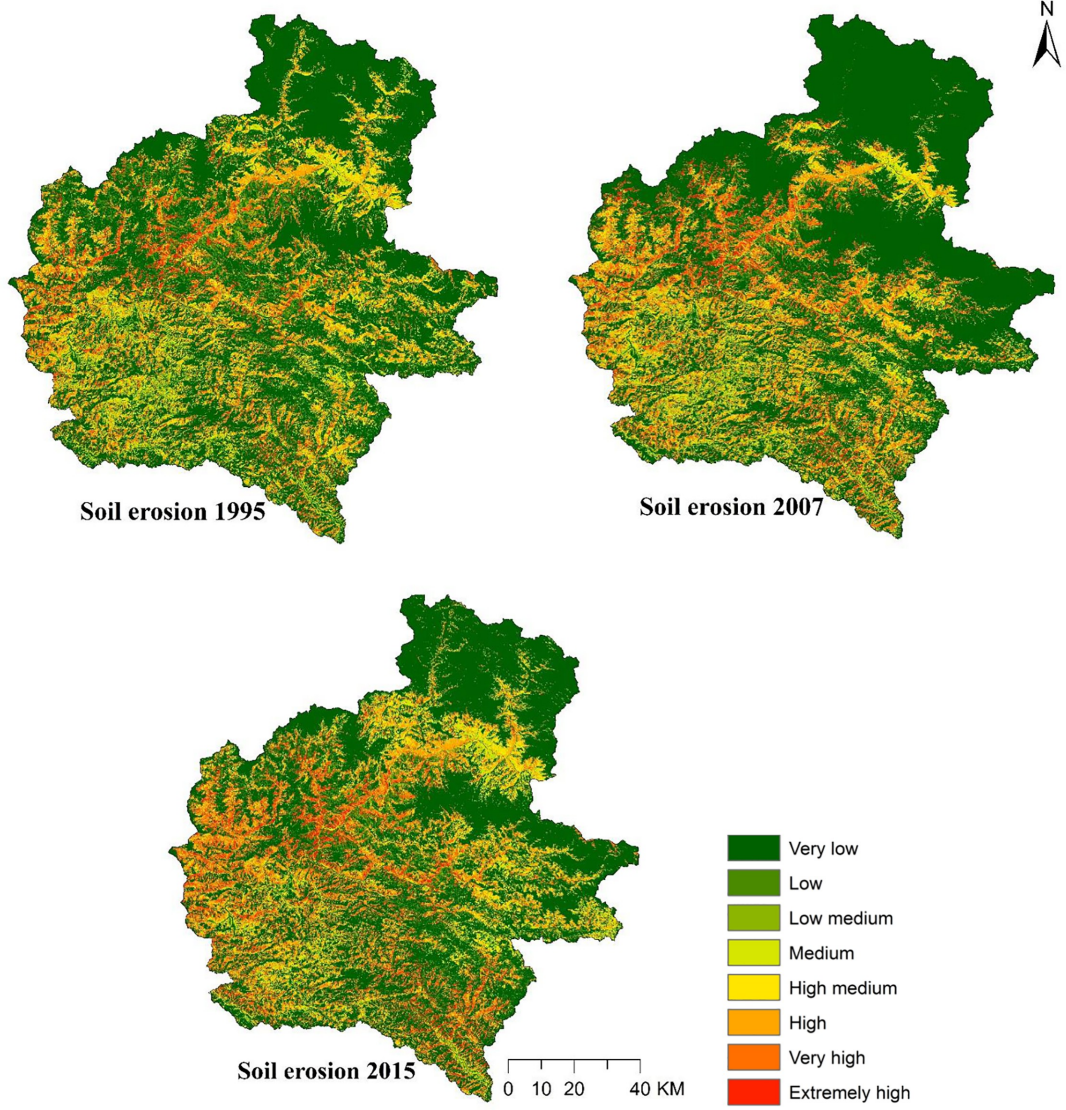
Soil erosion through the years 1995, 2007 and 2015.

The spatial distribution of *R*, *K*, *LS*, *C* and *P* factors are given in Figs 6 and 7, whereas the erosion maps for the years 1995, 2007 and 2015 are presented in Fig 8. As expected, soil erosion had increased through the years; mean erosion rate of 5.35 t/ha/year in 1995 had increased to 5.47 and 6.03 t/ha/year in 2007 and 2015, respectively. Soil erosion rates in the study area were regrouped into eight severity classes, and the respective areal coverage was presented according to guidelines developed by Uddin, Abdul Matin (9) (Table 6). Approximately 18% of the study area was severely eroded while the remaining 82% was least eroded in 1995. Similarly, areas under severe and less severe erosion zones were 18% and 82% in 2007, and 21% and 79% in 2015, respectively. It is important to note that we summed up the very low, low, low medium, medium and high medium classes as less severity, and high, very high and extremely high classes as high severity for soil erosion in the study area. Overall, the highly eroded area increased by 20% while the area under less severity decreased by 4% in the study area during 1995–2015.

**Table 6:** Soil erosion classes and severity in the study area through the years 1995, 2007 and 2015.

Soil loss (t/ha/year)	Erosion severity	1995		2007		2015	
		Area (km^2^)	%	Area (km^2^)	%	Area (km^2^)	%
0–0.5	Very low	7168.23	58.84	7459.5	61.24	6906.79	56.7
0.5–1	Low	685.89	5.63	397.98	3.26	430.22	3.53
1–2	Low medium	305.43	2.51	288.4	2.36	239.84	1.96
2–5	Medium	787.74	6.46	808.5	6.64	747.17	6.13
5–10	High medium	1067.51	8.76	1046.02	8.59	1252.23	10.28
10–20	High	1085.1	8.91	1044.01	8.56	1375.69	11.29
20–50	Very high	829.64	6.82	901.56	7.42	1012.31	8.32
> 50	Extremely high	252.76	2.07	236.33	1.93	218.05	1.79

Soil loss was the greatest in the Siwalik Hills with rates of 8.77, 8.99 and 9.84 t/ha/year in 1995, 2007 and 2015, respectively (Table 7), and the erosion rates were low in the High Himalayas with values of 1.10, 0.07 and 0.86 t/ha/year in1995, 2007 and 2015, respectively. Increasing soil erosion rates through all the physiographic zones, except the High Himalayas, were experienced over 21 years.

**Table 7:** Variation of soil loss with physiographic zones.

Physiographic zones	Total area		Mean erosion (t/ha/year)		
	km^2^	%	1995	2007	2015
Terai Plains (516–700 m)	98.81	0.82	3.30	3.34	4.46
Siwalik Hills (700–1500 m)	2535.39	20.81	8.77	8.99	9.84
Middle Mountains (1500–2700 m)	4281.47	35.14	7.66	8.24	8.26
High Mountains (2700–4000 m)	2909.09	23.88	4.87	4.39	5.59
High Himalayas (4000–7264 m)	2357.54	19.35	1.10	0.07	0.86

### Effect of LULC on soil loss

Erosion was the most severe in the agricultural lands in all of the target years: 26.46, 23.56 and 21.54 t/ha/year for 1995, 2007 and 2015, respectively (Table 8), followed by the bare lands with an average soil loss rate of 8.3 t/ha/year. The increase in the built-up areas and their expansion up to steep slopes and higher elevations led to an increase in soil erosion in the built-up area by 11.05 t/ha/year in 2015 as compared to 0.05 t/ha/year in 1995. Soil loss was very little in the forestlands as compared to other land uses, being just 0.3 t/ha/year throughout the study period. With 97–98% of contribution to gross soil erosion, agriculture and bare lands were the most significant source of soil sediments in the study area. The contribution of forests to gross soil erosion was 1.86, 1.47 and 1.61% in 1995, 2007 and 2015, respectively.

**Table 8:** Variation of soil loss with LULC.

Land use	1995				2007				2015			
	Area (km^2^)	Mean erosion rate (t/ha/year)	Total soil loss (t/year)	Contribution (%)	Area (km^2^)	Mean erosion rate (t/ha/year)	Total soil loss (t/year)	Contribution (%)	Area (km^2^)	Mean erosion rate (t/ha/year)	Total soil loss (t/year)	Contribution (%)
**Agriculture**	1780.78	26.46	47119.43	64.06	2178.56	23.56	51326.87	71.72	2722.3	21.54	58638.34	72.66
**Bare land**	2994.56	8.37	25064.46	34.08	2504.96	7.64	19137.89	26.73	2311.5	8.91	20595.46	25.51
**Built-up area**	0.2	0.05	0.01	0.0001	7.1	8.11	57.58	0.08	16.1	11.05	177.90	0.22
**Forest**	4695.26	0.29	1361.62	1.86	3518.04	0.3	1055.41	1.47	4170.76	0.31	1292.93	1.61
**Snow**	2673.3	0	0	0	3942.44	0	0	0	2946.44	0	0	0
**Water bodies**	38.2	0	0	0	31.2	0	0	0	15.2	0	0	0
**Total**	12182.3	5.35	73545.52	100	12182.3	5.47	71577.75	100	12182.3	6.03	80704.63	100

## Discussion

The observed changes in the different LULC classes throughout 1995, 2007 and 2015 can be explained with one of the following reasons. First, the increase in population by 43% in 2011 as compared to 1991 [49] had significantly increased the built-up areas during the 1995–2015 period. Increase in the agricultural area (52.87%) at the expense of bare lands and forests through the years can also be attributed to the population rise. Second, increasing awareness of community-based forestry proved its significance in protecting forests throughout the country [50]. Even though an overall decrease of 11.17% in the forests was observed during the study period, there was a rise in the forest area by 18.55% during the 2007–2015 period (Table 4); similar results have been reported by Uddin, Abdul Matin (9) while looking at the LUCC during 1990–2010 period for the whole area of Nepal. The advent of the Community Forest User Group in 1993 and the inclusion of multi-stakeholders in decision-making processes since 2000 [50] have become a substantial success in protecting forests throughout the country, and the study area is no exception regarding this. Overall, agricultural lands, built-up area and snow cover had increased, resulting in the subsequent decline in the bare lands, forests and water bodies. The increasing population had increased the built-up areas, but the migration of people abroad or to urban centres in search of better job opportunities have left some agricultural areas uncultivated. Land Use Policy (2013, 2015) has banned the illegal conversion of one land use to another [51]; however, partitioning and illegitimate conversion of agricultural lands for commercial purposes are becoming a major issue for the society. This practice also exacerbates soil loss.

An inspection of the change matrix (Table 5) shows an increase in the agricultural areas at the expense of bare lands and forests during the 1995–2007 period. This change has led to an increase in the soil erosion in the area for the described period as the erosion rates are higher in the agricultural lands in comparison to the bare lands and forests. Similarly, a decrease in the bare lands with a corresponding increase in the forests (15%) and agricultural lands (8%) was reported for the same period. Since the erosion rates in the forests were meagre (0.3 t/ha/year), increase in the forests contributed very little to the decrease in soil loss, whereas the agricultural lands having higher erosion rates significantly increased soil loss during the 1995–2007 period. Again, an increase in the agricultural lands with a corresponding decrease of bare lands (38%) and forests (8%) was observed during 2007–2015. Snow cover had reduced by 2015, which also had a marked increase in soil erosion during the period. An overall increase in the agriculture, built-up areas and snow cover and decrease in the bare lands, forests and water bodies were observed during 1995–2015. Soil erosion rates in the forest, water bodies and snow were negligible. Although with higher erosion rates, built-up areas occupied less area, so the contribution of the built-up area in determining the rate of soil loss was also negligible. Thus, it can be concluded that the increase in the agricultural lands at the expense of bare lands and forests escalated soil loss in the area.

Not only the erosion rates have increased through the years, but also an increase in severely eroded area by 20% and a decrease in the less severe area by 4% were reported. Soil erosion was the highest in the Siwalik Hills, ranging from 8.77 to 9.84 t/ha/year, and the lowest in the High Himalayas (0.07 to 1.10 t/ha/year). Presence of erodible silt particles and occurrence of steep slopes have made the Siwalik Hills the most eroded zone across the study area. The second most eroded zone was the Middle Mountains followed by the High Mountains, Terai Plains and High Himalayas. Gently sloping flatlands in Terai Plains [52] and presence of snow cover in the High Himalayas make these physiographic regions less susceptible to soil erosion.

Agriculture was the most eroded land use in the study area. Lack of vegetation cover is the main reason for higher soil losses in the agricultural lands [53]. Erosion rates through agricultural fields had reduced through the years, but with the upsurge in agricultural lands, the contribution of agriculture to gross soil erosion ultimately increased. Surprisingly, bare lands lost soil at lower rates than the agricultural fields; this may be due to the continuous and excessive tillage in the crop fields [14] while soil may have stabilized or compacted to resist the soil erosion to some extent in the bare lands. Community-based forestry showed positive impacts in safeguarding the forest resources after 2007.

The RUSLE has normally been utilized to estimate soil erosion in gentle slopes across the world. However, it has been increasingly used to assess soil loss across all the slopes ranging from gentle to steep slopes [54, 55]. The RUSLE has been extensively used in Nepal as well to calculate the erosion rates in areas with steep slopes too [9, 40, 56].

Although the results come with several future possibilities, this method has some limitations too. Some improvements can be made in the calculation of the RUSLE factors. In this research, annual rainfall data were used to calculate the *R* factor, whereas more intense rainfall events which are more likely to have a marked impact on soil loss [40] were not considered. Erosion potential of a given rainstorm is calculated by multiplying kinetic energy of the rain with maximum 30-minute rainfall intensity. Since the meteorological stations of Nepal do not have adequate laboratory facilities to measure 30-minute rainfall data, they were not considered in calculating the *R* factor. However, there is a possibility of improving the *R* factor in the future. Similarly, better estimates of the *K* factor can be obtained if soil texture (sand, silt and clay %) and soil organic matter data are available instead of just the soil textural classes. It also lacks the automation of the procedure in calculating the RUSLE factors.

It would be much useful to verify the soil erosion estimates with real soil loss observations from long term soil erosion plots. However, the availability of only a few soil erosion location data restrained us from validating the estimates and, thus, analyzing the errors further. We compared the soil erosion rates computed from the RUSLE with plot-based erosion measurements from another experiment conducted in the study area. The two-year data from the field plots produced annual soil loss rates in the range of 9–10 t/ha/year (approx.) which is higher than the RUSLE derived soil loss. We confirmed the synergistic interaction of mulch with tillage to lower the losses of soil organic matter and total nitrogen and the effectiveness of no-till to reduce the soil losses in the study area as well. Erosion plots include both the corn and bare fields; where all the plots except the bare ones received tillage/no-tillage and/or mulching/no mulching practice. The mean soil loss in the study area will be lower than what is reported in the erosion plots as a large part of the study area are forests, and soil loss will be much lower in the forests.

This study used a modelling approach to develop spatial distribution maps of water erosion for 1995, 2007 and 2015 using the RUSLE, GIS and Remote Sensing. Spatial distribution of soil erosion maps, such as produced here, can be a vital tool for planning, particularly where the local economy is mainly agriculture-based and rapid urban development has taken over the agricultural lands [57], as in this instance.

## Conclusions

This study provided information on the changes in LUCC in Sarada, Rapti and Thuli Bheri river basins of Nepal over the 1995–2015 period and analysed the effects of these changes on the rates of soil loss in the study area. The RUSLE was used in conjunction with GIS and Remote Sensing taking three temporal Landsat imageries from 1995, 2007 and 2015. The results suggest that the long term LUCC has a significant impact on soil loss in the study area as soil loss rates have increased over time. Increase in the agricultural lands at the expense of forests and bare lands have increased soil erosion. Of the different land uses, erosion was the highest in the agricultural lands. Forest cover seemed much more effective in reducing soil loss, even at the steeper slopes too. Soil erosion rates were the highest in the Siwalik Hills and Middle Mountains as compared to other physiographic regions. Proper agricultural management is needed to reduce the soil loss in the agricultural lands, focusing more on the steeper slopes.

The use of the RUSLE, GIS and Remote Sensing to estimate the soil erosion dynamics over other soil erosion estimation models has many advantages as it saves a substantial amount of time [12] and also avoids tedious measurements of soil sediments over time. The analysed land use maps and estimated soil erosion severity maps may be helpful for policymakers and planners in developing better soil and water conservation programs across the country. Since LUCC is inevitable in the future, sustainable land use plans should be formulated to keep soil erosion rates under control so that it does not pose added threats to the ecological sustainability of the area.

## Supporting information

S1 File Rainfall data from 53 rainfall stations (1990–2016). (CSV)
